# Tracking Virus-Specific CD4+ T Cells during and after Acute Hepatitis C Virus Infection

**DOI:** 10.1371/journal.pone.0000649

**Published:** 2007-07-25

**Authors:** Michaela Lucas, Axel Ulsenheimer, Katja Pfafferot, Malte H.J. Heeg, Silvana Gaudieri, Norbert Grüner, Andri Rauch, J. Tilman Gerlach, Maria-Christina Jung, Reinhart Zachoval, Gerd R. Pape, Winfried Schraut, Teresa Santantonio, Hans Nitschko, Martin Obermeier, Rodney Phillips, Thomas J. Scriba, Nasser Semmo, Cheryl Day, Jonathan N. Weber, Sarah Fidler, Robert Thimme, Anita Haberstroh, Thomas F. Baumert, Paul Klenerman, Helmut M. Diepolder

**Affiliations:** 1 Peter Medawar Building for Pathogen Research, Nuffield Department of Clinical Medicine, University of Oxford, Oxford, United Kingdom; 2 Medical Department II and Institute for Immunology, Ludwig-Maximilians-University Munich, Munich, Germany; 3 Centre for Clinical Immunology and Biomedical Statistics, Royal Perth Hospital and Murdoch University, Perth, Australia; 4 Centre for Forensic Science, School of Anatomy and Human Biology, University of Western Australia, Nedlands, Australia; 5 Division of Infectious Diseases, University Hospital, Berne, Switzerland; 6 Clinica delle Malattie Infettive, University of Bari, Bari, Italy; 7 Max von Pettenkofer-Institute, Department of Virology, Ludwig-Maximilians-University Munich, Munich, Germany; 8 Department of Medicine, Imperial College, St. Mary's Hospital, London, United Kingdom; 9 Albert-Ludwigs-Universität, Freiburg, Germany; 10 Inserm Unité 748, Université Louis Pasteur, Strasbourg, France; National Institute for Communicable Diseases, South Africa

## Abstract

**Background:**

CD4+ T cell help is critical in maintaining antiviral immune responses and such help has been shown to be sustained in acute resolving hepatitis C. In contrast, in evolving chronic hepatitis C CD4+ T cell helper responses appear to be absent or short-lived, using functional assays.

**Methodology/Principal Findings:**

Here we used a novel HLA-DR1 tetramer containing a highly targeted CD4+ T cell epitope from the hepatitis C virus non-structural protein 4 to track number and phenotype of hepatitis C virus specific CD4+ T cells in a cohort of seven HLA-DR1 positive patients with acute hepatitis C in comparison to patients with chronic or resolved hepatitis C. We observed peptide-specific T cells in all seven patients with acute hepatitis C regardless of outcome at frequencies up to 0.65% of CD4+ T cells. Among patients who transiently controlled virus replication we observed loss of function, and/or physical deletion of tetramer+ CD4+ T cells before viral recrudescence. In some patients with chronic hepatitis C very low numbers of tetramer+ cells were detectable in peripheral blood, compared to robust responses detected in spontaneous resolvers. Importantly we did not observe escape mutations in this key CD4+ T cell epitope in patients with evolving chronic hepatitis C.

**Conclusions/Significance:**

During acute hepatitis C a CD4+ T cell response against this epitope is readily induced in most, if not all, HLA-DR1+ patients. This antiviral T cell population becomes functionally impaired or is deleted early in the course of disease in those where viremia persists.

## Introduction

Hepatitis C virus (HCV) infection is a major cause of chronic liver disease worldwide and is thought to affect over 170 million people [1]. The virus readily sets up persistent infection in the majority of those who acquire it, evading host innate and adaptive immunity. A smaller proportion of those infected, however, do spontaneously control virus infection–in these, cellular immune responses are thought to play an important role [2], [3]. Functional assays and genetic studies point to a prominent role of HCV-specific CD4+ and CD8+ T cells in this initial control, as well as innate responses driven by NK cells and neutralising antibodies [4]–[6]. Animal models of HCV demonstrate a key role for CD4+ T cells, especially upon rechallenge [7]. Until recently, direct evaluation of virus-specific CD4+ T cells has not been possible, except in transgenic systems. However, recent advances in HLA class II tetramer technology and improvements in staining techniques have allowed CD4+ T cell populations to be observed ex vivo in a variety of settings, including HCV, influenza and HIV [8]–[11]. These tools are based on the refolding of soluble, biotinylated HLA class II molecules with specific viral peptides in vitro, and tetramerisation around fluorescently labelled streptavidin molecules to allow strong binding of the complex with the T cell receptor of the specific cells present within a population. Typically these cells occur at low frequencies, 1-2 logs lower than those detected with class I-peptide tetramers, which bind CD8+ T cells. Using magnetic bead enrichment techniques, however, the HLA class II tetramer staining can reliably detect populations of specific CD4+ T cell as small as 0.001% of total CD4+ T cells (corresponding to 10/10^6^) [9], [12]–[14].

Here we present the first longitudinal study with HLA class II tetramers in patients with different outcomes of acute hepatitis C. We took advantage of an unusually immunodominant, HLA-DR1 restricted CD4+ T cell epitope which we had characterized in detail by specific CD4+ T cell clones from three different patients and which was complexed into an HLA-DR1 tetramer. This novel tetramer was used to explore the frequency, phenotype and function of hepatitis C virus (HCV)-specific CD4+ T cells in seven patients with acute hepatitis C during the most critical phase of viral elimination or persistence. We identified a novel pattern of dynamics of helper cell populations associated with viral persistence, which is likely to be critical in the evolution of HCV towards persistent infection.

## Materials and Methods

### Patients

Seven HLA-DR1 positive patients with acute hepatitis C were included in this study. All patients were available for study within the first 3 months after onset of symptoms. Acute hepatitis C was diagnosed by documented seroconversion to anti-HCV antibodies or all of the following: acute onset of hepatitis in previously healthy individual, aminotransferases at least 10× the upper limit of normal, exclusion of other infectious, metabolic, or toxic causes of hepatitis, recent exposure or source of infection identified. The clinical characteristics of the patients are summarized in Table 1.

**Table 1:** Clinical data of patients with acute hepatitis C

Patient	Sex	Route of infection	Therapy	Outcome	Genotype
*Acute-Resolver (mean age 44.5 years)*					
AR1	F	hospital	None	Resolved	nd
AR2	F	unknown	IFN+RBV	SR	1a
*Acute-Chronic (mean age 26.8 years)*					
AC1	F	i.v. drugs	None	Chronic	1b
AC2	M	hospital	None	Chronic	1b
AC3	M	i.v. drugs	IFN+RBV	SR	1b
AC4	F	unknown	IFN+RBV	SR	nd
*Acute–lost to follow-up*					
AX	F	i.v. drugs	None	unknown	nd
*Long-term Resolver (mean age 32 years)*					
R1	F	sexual	None	Resolved	1a
R2	F	blood product	None	Resolved	1
*Chronic hepatitis C (mean age 42 years)*					
C1	M	unknown	None		1
C2	M	unknown	None		1b
C3	M	unknown	None		2
C4	F	i.v drugs	IFN+RBV	NR	3a
C5	F	unknown	None		1b

IFN = interferon-alpha; RBV = ribavirin; NR = non-responder to antiviral therapy; SR = sustained responder to antiviral therapy.

Five HLA-DR1 positive patients with established chronic hepatitis C (HCV-RNA positive, anti-HCV positive) and two long-term recovered patients (anti-HCV positive, HCV-RNA negative) were studied for CD4+ T cell responses in peripheral blood (Table 1). In six other HLA-DR1 positive patients with chronic hepatitis C liver derived T cell lines were analysed; the clinical details of these have been reported previously [15].

Four healthy and four HIV-infected HLA-DR1-positive individuals, as well as two healthy and five acutely HCV-infected HLA-DR1-negative individuals (n = 15) served as controls.

All patients gave informed consent to participate in the study and the protocol and the procedures of the study were conducted in conformity with the ethical guidelines of the Declaration of Helsinki. The patients with acute and chronic hepatitis C were recruited at the University Hospital Munich or at the University of Freiburg (for intrahepatic T cell lines [15]) according to study protocols approved by the local ethical committee. The sequence data are derived from the Genetic Cohort of the Swiss HIV Cohort study (www.shcs.ch) that have all given informed consent for use of clinical data, viral sequencing and genetic analyses. The HIV positive controls were recruited from an ethically approved study of St Mary's Hospital, London, UK (protocol 99/IA/161E).

### HCV proteins and peptides

Recombinant HCV proteins were kindly provided by M. Houghton (Chiron, Emeryville, CA) comprising the non-structural protein 4 (NS4) or/and the non-structural protein 3 (NS3) region of the HCV polyprotein (c100, c200). Proteins were expressed as COOH-terminal fusion proteins with human superoxide dismutase in yeast (Saccharomyces cerevisiae). Protein was >90% pure.

Eighty-three overlapping peptides (20mers) covering the region amino acid (aa) 1207–2014 were synthesized by Chiron Mimotopes (Clayton, Australia) and 301 overlapping peptides (15mers) covering the entire HCV polyprotein were synthesized by EMC (Microcollections, Tübingen, Germany).

### Proliferation assay

Peripheral blood mononuclear cells (PBMC) were isolated on Ficoll-Hypaque gradients (Biochrom, Berlin, Germany) and washed four times in phosphate buffered saline (PBS). PBMCs (5×10^4^ cells/well) were incubated in 96-well U-bottom plates (TPP, Trasadingen, Switzerland) for 5 days in the presence of HCV proteins (2 µg/ml; c100) in 150 µl of tissue culture medium (RPMI 1640 medium; Biochrom, Berlin, Germany) containing 2 mM L-glutamine, 1 mM sodium pyruvate (Serva, Heidelberg, Germany), 100 U of penicillin (Biochrom, Berlin, Germany) per ml, 100 µg of streptomycin (Biochrom, Berlin, Germany) per ml and 5% human AB serum (Pan, Aidenbach, Germany). Cultures were labelled by incubation for 16 h with 2 µCi of 3H-thymidine (specific activity, 80 mCi/mmol; Amersham, Little Chalfont, United Kingdom). Triplicate cultures were assayed routinely, and the results expressed as mean counts per minute. The stimulation index (SI) was calculated as the ratio of counts per minute obtained in the presence of antigen to that obtained without antigen. A SI of >3 was considered significant.

### Elispot assay

Nitrocellulose-bottom 96-well millititer HA plates (Millipore, Bedford, MA) were coated with 100 µl of IFN-gamma monoclonal antibody (Mabtech, Stockholm, Sweden), at a concentration of 15 µg/ml in PBS. Plates were incubated at 4°C overnight; before use unbound antibody was removed by excessive washing with PBS. The coated wells were filled with 100 µl tissue culture medium containing 2×10^5^ freshly isolated PBMC and NS4 protein (c100, 2 µg/ml). After 48 h incubation, cells were washed off and the biotin-conjugated anti-IFN gamma antibody (Mabtech, Stockholm, Sweden, 1 µg/mL) was added. After an incubation period (4 h, room temperature) unbound antibody was washed off and 100 µl of the streptavidin-alkaline phosphatase (Mabtech, Stockholm, Sweden) was added for 1 h. Unbound conjugate was washed off and, finally, 100µl of 5-bromo-4-chloro-indolylphosphate/nitroblue tetrazolium substrate solution (Bio-Rad Lab., Richmond, CA) was added for 2 h. The colour reaction was stopped by extensive washing. After drying the number of spots was scored by use of a dissection microscope.

### MHC II tetramer staining

The DRB1*0101 tetramer complexed with HCV 1806-1818 (TLLFNILGGWVAA) was custom synthesized by Beckman Coulter Immunomics (France, Marseille). PBMC were stained in 100 µl medium (RPMI, 10% fetal calf serum, 2 mM glutamine, 50 U/ml Penicillin-Streptomycin) with 3 µl of Phycoerythrin (PE)-conjugated MHC class II tetramer for 2 hours at room temperature. Allophycocyanin-conjugated anti-CD4, Peridinin Chlorophyll-*a* Protein-conjugated anti-CD14, Peridinin Chlorophyll-*a* Protein-conjugated anti-CD19, Viaprobe (Becton Dickinson), and FITC-conjugated anti-CD38 monoclonal antibody (Becton Dickinson) were added after at least 20 minutes of incubation. Cells were washed twice and then incubated with anti-PE microbeads for 20 minutes at 4°C. Cells were then washed once and 90% of cells were applied to MS columns (Miltenyi Biotec, Bergisch Gladbach, Germany) according to the manufacturer̀s instructions. The other 10% were reserved for FACS analysis. The PE positive cells were eluted from the column (post-enrichment sample) and analyzed by FACS. Cells were gated on the CD4+, CD14-, CD19- and Viaprobe-population.

### Calculation of tetramer-positive T cell frequencies and validation of the method

Frequencies of tetramer-positive CD4+ T cells were determined as described previously [9], [12]–[14]. Briefly, the input number of CD4+ T cells in the enriched sample (90% of total) was calculated by multiplying the number of CD4+ cells in the pre-enrichment sample (10% of total) by a factor of 9. The frequency of tetramer-positive cells was thus calculated by dividing the post-enrichment output number of tetramer-positive cells by the calculated input number of CD4+ cells.

### Expansion of liver infiltrating lymphocytes

Biopsies were obtained in patients with chronic hepatitis as clinically indicated. From material not needed for histological analysis, lymphocytes were isolated and expanded as explained previously [15]. In brief, homogenized cell suspensions were incubated with magnetic beads coupled to anti- CD4 antibodies, and bound cells were isolated using a particle magnetic concentrator. The purity of the T-cell subset, which was confirmed by fluorescence-activated cell sorting (FACS) analysis, was always greater than 90%. The isolated intrahepatic T cells were then plated into separate wells in 24-well plates (Greiner, Essen, Germany) in 1 mL 10% human AB serum, 100 U/mL IL-2 (Hoffmann–La Roche, Basel, Switzerland), 0.04 µg/mL anti-human CD3 monoclonal antibody (Immunotech, Marseilles, France), and 2×10^6^ irradiated autologous PBMCs as feeder cells.

### Sequencing

Viral RNA was extracted from plasma samples using either the QIAamp Viral RNA Mini Kit (QIAGEN) or the COBAS AMPLICOR HCV Specimen Preparation Kit v2.0 (Roche) according to manufacturer's instructions. An initial RT-PCR using the primers 2412F2 (CACCTCCACCARAACATYGT) and 9192R (GGAGTGAGTTTGAGCTTGGT) in combination with the SuperScript III One-Step RT-PCR System with Platinum Taq DNA polymerase PCR Kit (Invitrogen) was performed. The first-round product was then used in nested 2nd round PCRs to amplify the relevant NS4 region with the following primer pairs, HCV-5040F/HCV-HCV-6121R (CACATAGATGCCCACTTCCT/GCCCCGGGAGGCRAASGC) and HCV-6076F/HCV-7636R (CTGTGCARTGGATGAACMG/TGATGAGGGCSCCKGTCCA) or alternatively the genotype 1-specific primers 5517M13F/6531M13R (GAGCARTTCAAGCAGAAGGCSCTC/AGGGGCCCGTGGTGTABGCGTT) together with the Platinum Taq DNA Polymerase High Fidelity Kit (Invitrogen). PCR products were purified using the Exosap method (Amersham) and BigDyeTerminator (v3.1) sequencing reactions were set up with PCR or M13 primers. The samples were run on an ABI 3730 Genetic Analyser and electropherograms analysed using Seqscape v2.0 (ABI).

### HLA Typing

DNA was obtained from whole blood using the QIAamp DNA Blood Mini Kit following the manufacturer's guidelines. HLA Class II typing was performed by PCR and/or direct DNA sequencing using previously described methods [16].

## Results

### Validation of the HLA-DR1-HCV-1806-1818 tetramer and staining of specific CD4+ T cell clones

**Figure 1:**
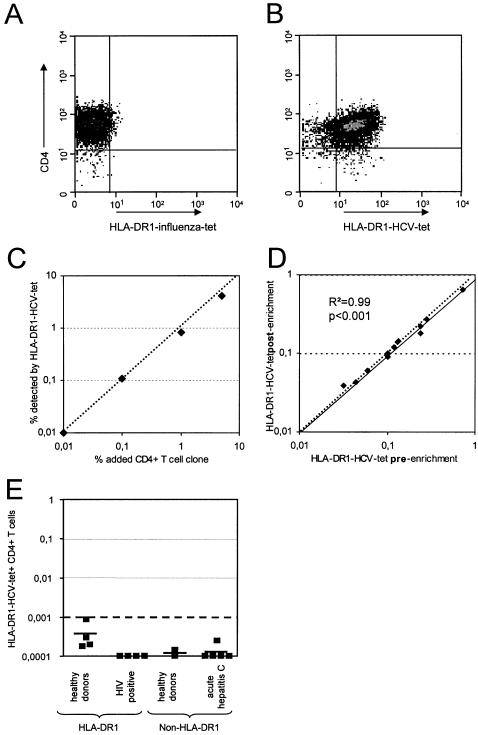
Validation of HLA-DR1-HCV-1806-1818 tetramer staining and enrichment technology. A CD4+ T cell clone specific for HCV 1808-1817 was stained with the HLA-DR1-HCV-1806-1818 tetramer and a control HLA-DR1 tetramer specific for the influenza epitope 307-319 and analysed by flow cytometry as described in the methods (A+B). The HCV clone was derived from patient AR1. Panel C shows staining of the clone diluted serially into PBMC negative for the tetramer at the concentrations shown. Panel D shows a strong correlation between observed pre-enrichment frequency and calculated post-enrichment frequency of HLA-DR1-HCV-tetramer positive CD4+ T cells in all PBMC samples with a frequency of HLA-DR1-HCV-tetramer positive CD4+ T cells of >0.03% (R^2^ = 0.99, p<0.001). The dotted line is the bisecting line, the solid line represents the trend line of the measured frequencies. Panel E shows the staining of control groups; four healthy and four HIV-infected HLA-DR1 positive individuals, and two healthy individuals and five acute hepatitis C patients without HLA-DR1.

For tetramer synthesis we selected the epitope HCV-1806-1818 which has previously been shown to be a highly targeted epitope in HLA-DR1 positive patients with acute or resolved hepatitis C [17], [18]. This HLA-DR1 tetramer strongly and specifically stained epitope specific CD4+ T cell clones from both HLA-DRB1*0101 and *0102 positive individuals (Figure 1A+B). Since the expected frequency of virus specific CD4+ T cells in PBMC was below 0.1% of CD4+ T cells we employed an enrichment technique using antibodies to PE coupled to magnetic beads that allows accurate quantification of rare populations of antigen-specific cells above 0.001% of input CD4+ T cells, corresponding to 10/10^6^ CD4+ T cells [9], [12]–[14]. To test the accuracy of the assay, we titrated specific clone cells into autologous PBMC which had been depleted of specific CD4+ T cells. Over a range of 0.01 to 5% epitope specific CD4+ T cells could reliably be enumerated (Figure 1C). The enrichment technique was validated in PBMC samples with a frequency of tetramer positive cells >0.03% (n = 12 time points), because in those samples a valid frequency determination could be performed both before and after enrichment. A high agreement was found with a R2 of 0.99 (p<0.001, Figure 1D).

In the controls a mean of 0.73±0.75 tetramer+ CD4+ T cells (range 0–2) were found among a mean of 0.5×10^6^ CD4+ T cells that were analysed, corresponding to a mean frequency of 0.00016±0.00017% of CD4+ T cells (Figure 1E).

**Figure 2:**
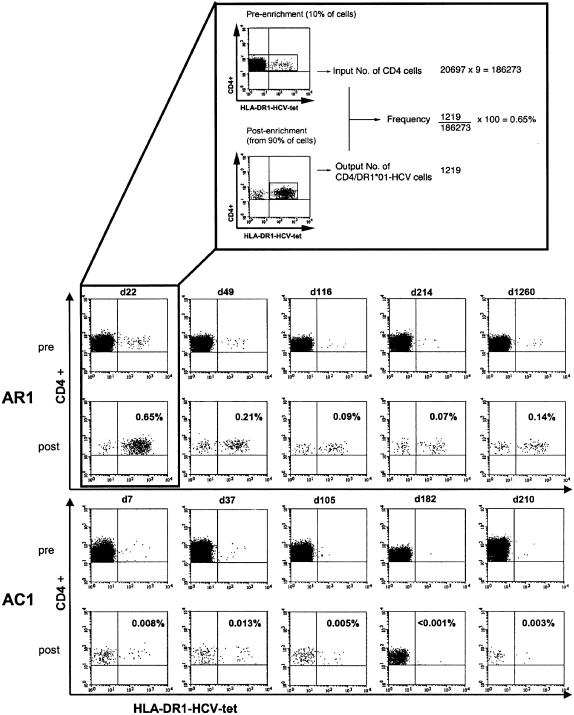
Examples of ex vivo HLA-DR1-HCV-1806-1818 tetramer staining from the acute hepatitis C cohort. PBMC from one acute resolving patient (AR1) and one patient with acute hepatitis C and chronic evolution (AC1) were stained with the HLA-DR1-HCV-1806-1818 tetramer as described in the methods. For each patient the upper panels represent pre-enrichment stainings and the lower-panels the post-enrichment stainings. The numbers in the upper right corner of the post-enrichment panels are calculated according to the input CD4+ T cell number. The insert on top of the figure shows in detail the calculation method. These figures are plotted over time in both cases in Figure 2 (lower panels).

Representative dotblots of the HLA class II tetramer staining in patients AR1 and AC1 before and after enrichment are shown in Figure 2 and an example of the frequency calculation is given in the figure insert.

### Acute study population and clinical course

**Figure 3:**
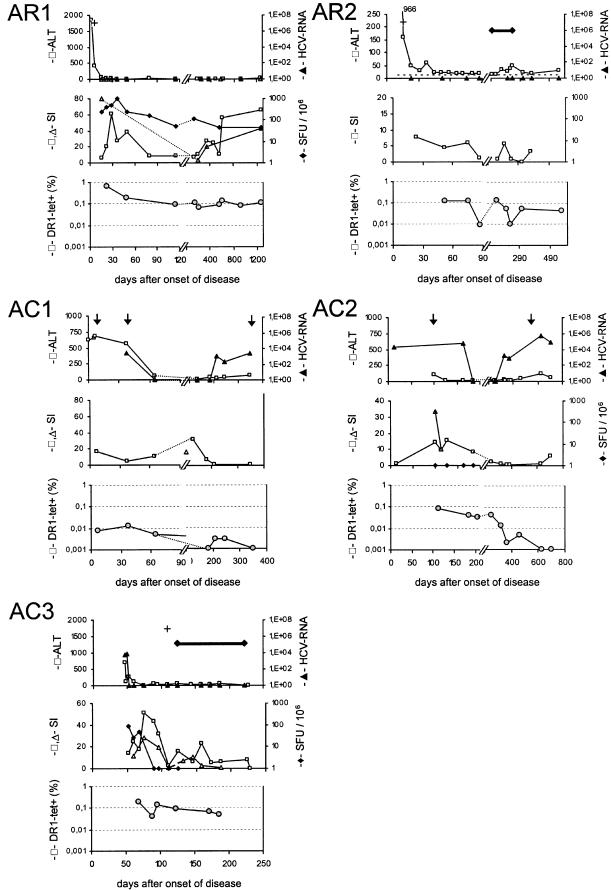
Clinical course of acute hepatitis C cohort in correlation to HCV specific CD4+ T cell response. The clinical course of each individual is shown in the upper panel in each case. Note the time scale (days) is different in each case. The upper panel shows the ALT (IU/L; open boxes; left hand y axis) and the serum HCV RNA level (closed triangles; right hand y axis). For AR1, AR2 and AC3, at some time points only qualitative assays for HCV-RNA were available which have been marked with a +. Interferon alpha therapy is marked with a bar. In patients AC1 and AC2 time points where the sequence of epitope 1806-1818 was determined are indicated by arrows. The middle panel in each case corresponds to functional CD4+ T cell assays: proliferation in response to NS3/4-antigen (open boxes) and peptide-1806-1818 (open triangles) is shown as stimulation index (SI) on the left hand y axis and HCV-antigen induced interferon-gamma secretion as determined by Elispot (solid diamonds) is shown as spot forming units (SFU) on the right y-axis. The lower panel of each case shows the frequency of HLA-DR1-HCV-1806-1818 tetramer positive CD4+ T cells (grey circles). The cut-off for detection was 0.001%. The upper limit of normal for ALT is indicated in panel AR2 as dotted line.

A cohort of seven HLA-DR1 positive persons with acute hepatitis C was available for study (Table 1). Five of those were sampled longitudinally from the first weeks of acute disease (median 4 weeks after onset of symptoms, range 1–90 days) for a mean follow-up of 21 months (range 6 to 42 months). One individual cleared spontaneously (AR1); another one had cleared spontaneously but received interferon-alpha treatment after three months despite negative HCV-RNA in the serum because of continuously elevated ALT levels and fear of relapse (AR2). Three of the cohort achieved transient viral control for 20, 21, and 6 weeks, respectively, before recrudescence of HCV-RNA in the serum (AC1-3). Of these, one patient (AC3) was treated successfully with interferon-alpha and ribavirin, one patient refused treatment (AC1) and patient AC2 had medical contraindications to antiviral treatment. The clinical course (ALT and viral load over time) of individuals AR1 and 2 and AC1-3 is shown for each person in Figure 3 (upper panel in each case).

The two other HLA-DR1 positive individuals with acute hepatitis C were available for study at a single time point one and four weeks after onset of disease, respectively. One was still viremic after 4 months and was treated successfully with antiviral therapy (AC4), and the other was lost to follow-up (AX).

### Epitope HCV-1808-1817 is highly targeted in our study cohort

**Figure 4:**
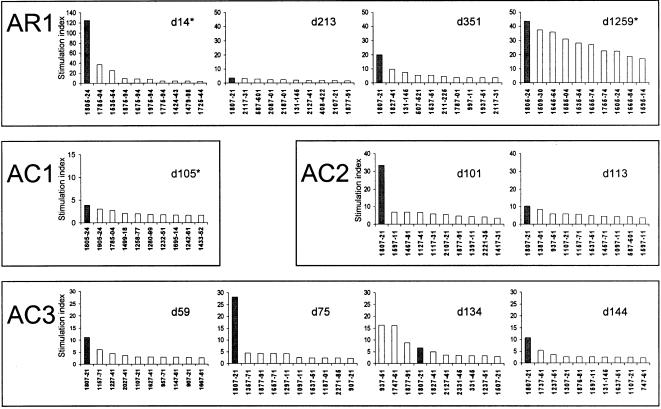
Relative immunodominance of epitope HCV 1805-24 in the acute HCV cohort. Ex vivo proliferation assays were performed on PBMC taken from the persons indicated over time. The responses to peptides including the sequence 1807-18 are marked with a dark bar. The ten peptides inducing the strongest proliferative response are shown in descending order with regard to the strength of the response. Asterisks indicate time points when the NS3-helicase/NS4 peptide library (covering aa 1207–2014) was used; all other assays were performed with a peptide library covering the entire HCV genome. A SI>3.0 was considered significant.

In four of the five longitudinally studied patients (AR1, AC1-3) proliferation assays with overlapping peptide libraries were performed to define the hierarchy of immunodominant epitopes (Figure 4). One peptide library was composed of 301 overlapping 15-mer peptides covering the entire HCV polyprotein, the other peptide library contained 83 overlapping 20-mer peptides covering NS3-helicase-NS4 (aa 1207–2014). The first library was used at eight time points (patients AR1, AC2, and AC3), the second library was used at three time points (patients AR1 and AC1). Importantly, in all patients the response to peptides containing the epitope present in the HLA-DR1-HCV-1806-1818 tetramer was strongest, indicating that the CD4+ T cell response against this epitope represents a major part of the overall HCV-specific T helper response in HLA-DR1 positive patients.

### HLA-DR1-HCV-1806-1818 tetramer staining

Tetramer+ CD4+ T cells were detected in all seven patients with acute hepatitis C, irrespective of clinical outcome, with frequencies ranging from 0.007% to 0.65% (mean 0.16%+/−0.2). Representative stainings from patients AR1 and AC1 showing both pre-enrichment and post-enrichment samples are displayed in Figure 2; the detailed correlation with ALT, viral load, HCV specific proliferation, and IFN-γ secretion of all five patients is shown in Figure 3. In the two patients with acute hepatitis C who were studied only at single time points (AX and AC4), the frequencies of tetramer+ CD4+ T cell were 0.05% and 0.007%, respectively.

In patient AR1, the highest frequency of tetramer+ CD4+ T cells (0.65% of peripheral CD4+ T cells) was measured at the earliest time point which was 2 weeks after spontaneous clearance of HCV-RNA from the serum. Tetramer+ CD4+ T cells subsequently declined to a level of about 0.1% and remained stable for more than 3.5 years of follow-up. Very strong peptide specific T cell proliferation and NS4-specific IFN-gamma secretion were present during the first 6 months after disease onset, and -despite some fluctuations-robust responses were maintained during long-term follow-up.

In patient AR2, relatively high levels of tetramer+ CD4+ T cells (0.12%) and significant T cell proliferation were present during the phase of viral clearance. At week 12 the frequency of tetramer+ CD4+ T cells declined by one log and proliferation was lost. Despite the continuing absence of HCV-RNA from the serum at that time, the constantly elevated ALT levels suggested a high risk of viral relapse and interferon-alpha treatment was initiated. The frequency of tetramer+ CD4+ T cells recovered to the initial levels and finally remained around 0.05% during the follow-up after antiviral therapy. T cell proliferation was detected only intermittently during and after interferon-alpha treatment.

All three patients with chronic evolution went through a phase of undetectable serum HCV-RNA in the first six months of disease. In all three, tetramer+ CD4+ T cells and significant antigen-specific proliferation were present during viral clearance and the first phase of viral control; antigen-specific proliferation, however, was completely lost in all three patients when viral relapse occurred. In patients AC2 and AC3 simultaneous IFN-γ Elispot assays revealed that IFN-γ secretion was absent even earlier. In patient AC3 this occurred three weeks before the loss of proliferation; in patient AC2 IFN-γ secretion could never be detected. Importantly, in AC2 and AC3 the loss of function occurred while the frequency of circulating tetramer+ CD4+ T cells was relatively stable at levels between 0.04% and 0.14%. While in patient AC2 the frequency of tetramer+ CD4+ T cells subsequently declined and eventually specific cells disappeared, successful interferon therapy in AC3 was associated with stable levels of tetramer+ CD4+ T cells although only a modest recovery of function was measurable. A different pattern was seen in patient AC1: here, the tetramer+ CD4+ T cells had already disappeared from the peripheral blood before viral recrudescence and only very low frequencies (0.003%) could be detected, intermittently, during the subsequent months. Importantly, in no case was the loss of HCV-specific CD4+ T cell function accompanied by a relevant decrease of recall antigen (tetanus toxoid) or mitogen (PHA) responses.

### Expression of the activation marker CD38 on tetramer+ CD4+ T cells

**Figure 5:**
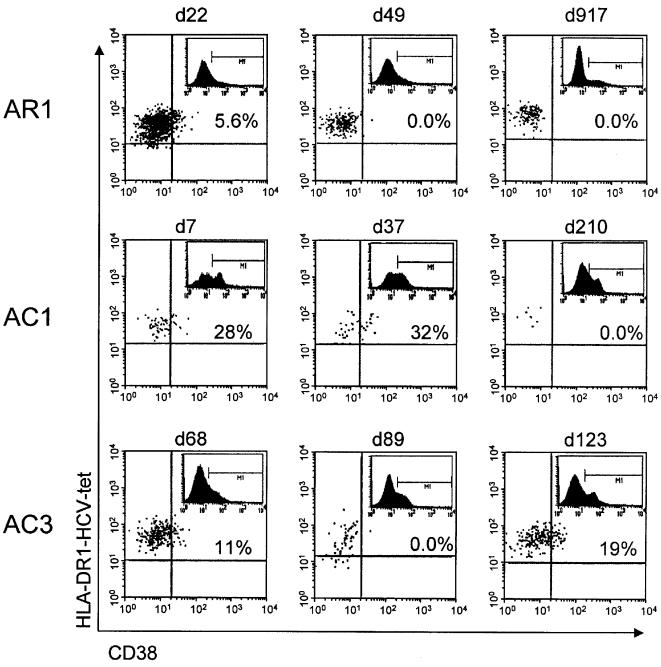
CD38 expression of HLA-DR1-HCV-1806-1818-tetramer positive CD4+ T cells. Longitudinal phenotypic analysis of the DR1 tetramer positive cells was performed for the activation marker CD38. The dot plots shown are gated on the DR1 tetramer positive cell population. Representative examples of the staining for AR1, AC1, and AC3 at early and late time points are shown and the percentage of CD38 positive cells is given for each plot. The inserted histograms show CD38 staining on all CD4+ T cells pre-enrichment and have been used to set the quadrants for the DR1 tetramer staining.

Longitudinal phenotyping for CD38 was routinely performed with all tetramer stainings; in the first samples that could be studied, between 5.8 and 32% of tetramer positive cells expressed CD38. In contrast, at all time points beyond 6 months, CD38 expression was negative in both recovered and chronically infected patients. Representative experiments are shown in Figure 5. In patient AR1 only 5.6% of tetramer+ CD4+ T cells were CD38 positive on day 22 which was 2 weeks after viral elimination, and well after the ALT peak. In all subsequent assays, CD38 was absent on tetramer+ CD4+ T cells. In contrast, in patient AC3 CD38 expression actually increased again to 19% when HCV-RNA re-appeared in the serum. The highest levels of CD38 expression on tetramer+ CD4+ T cells, 28% and 32%, respectively, were measured in patient AC1 just before tetramer+ CD4+ T cells were lost. Overall, we did not see very high levels of CD38 expression in this cohort, compared to studies using class I tetramers [4], [19].

### HLA-DR1-HCV-1806-1818 tetramer staining in patients with chronic hepatitis C and long-term resolvers

**Figure 6:**
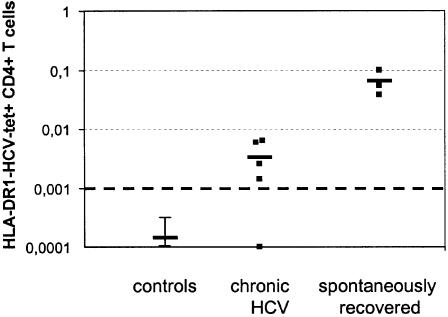
Ex vivo HLA-DR1-HCV-1806-1818 tetramer staining in stable resolved and chronic HCV infection. Tetramer staining was performed in five HLA-DR1-positive patients with chronic hepatitis C in comparison to three HLA-DR1-positive resolved patients. In all PBMC from control groups (five individuals with acute HCV who did not possess HLA-DR1, and four healthy and four HIV1+ individuals with HLA-DR1, respectively), tetramer staining was <0.001%. In all patients and healthy controls, proliferation assays following stimulation with recombinant NS4-antigen were performed. The SI for healthy controls was 1.2±0.48 (mean±SD, range 0.71 to 1.66), for chronic hepatitis C patients 1.7±0.9 (range 0.76 to 3.7), and for recovered patients 22.1±22.1 (range 1.74 to 52.9).

In HLA-DR1 positive patients with chronic hepatitis C a mean of 0.0032%+/− 0.0025 of CD4+ T cells stained tetramer+ (n = 6; range <0.001% to 0.0063%), which is more than 1.5 logs below the mean frequency of tetramer+ CD4+ T cells in patients in the early phase of acute hepatitis C, although still in some cases higher than the control stainings (DR1+HCV- and DR1-HCV+) which were all <0.001% (mean 0.00017%; Figure 6). In contrast, three patients with a history of spontaneous HCV clearance, including patient AR1 three years after viral clearance, maintained mean levels of tetramer+ CD4+ T cells of 0.064%+/− 0.03 (range 0.038% to 0.1%) for up to 20 years after acute hepatitis C.

One possible explanation for the results seen would be that virus-specific CD4+ T cells are compartmentalised entirely in the liver, once persistent infection is established. We therefore studied CD4+ T cells from six liver biopsies taken from chronically infected HLA DR1+ patients in parallel with peripheral blood samples. In no case was a detectable tetramer+ population seen (data not shown) whereas in parallel, using similar antigen-independent stimulation techniques, in the same cohort of patients substantial class I tetramer+ CD8+ T cell populations were visualised [15]. It is, however, conceivable that HCV-specific CD4+ T cells–in contrast to CD8+ T cells–are lost in vitro during the expansion step and further studies with an optimised protocol for direct ex-vivo HLA class II tetramer staining of liver infiltrating lymphocytes will need to be performed to resolve this issue.

### Analysis of immune selection within the HLA DR1 immunodominant epitope

Another explanation for the clear loss of tetramer+ populations, and loss of responsiveness associated with chronic infection would potentially be immune escape within the target epitope. To address this question, we sequenced early and late viral samples from patients AC1 and AC2. In patient AC3 no HCV sequence could be obtained probably due to the low viral load before antiviral therapy was initiated. In both early and late samples the sequence within epitope 1805-1824 corresponded to the prototype sequence (Table 2). To further expand this data we added a population-based approach, as has been used in the evaluation of CD8+ T cell responses to HCV restricted by HLA-B8 [20] and B27 [21]. We analyzed the sequences in 8 HLA DR1+ individuals with stable chronic infection, from cohorts in Munich, Australia and Switzerland and compared this to sequences of 80 HLA DR1- controls. The data are displayed in Table 3, which reveals a very limited degree of diversity in this epitope, and a mutation seen in only 1 out of 8 of the DR1+ patients, a frequency which does not differ from that in the HLA DR1- control group (10 of 80, p = n.s.). Given the strong conservation of this peptide sequence, it seems unlikely that immune escape through mutation is a major contributor to the decline in T cell responses observed, unless it is only transient and followed by reversion. Interestingly, while this epitope is highly conserved within genotype 1 sequences, there is less sequence conservation if compared to other genotypes (http://www.bioafrica.net/GDElinux/GDEmicrobial.html).

**Table 2:** Sequence evolution of epitope 1806-1818 in acute hepatitis C.

Pt.	Time point	Sequence aa 1806-1818
AC1		
	day 7	TLLFNILGGWVAA
	day 37	-------------
	day 347	-------------
AC2		
	day 101	TLLFNILGGWVAA
	day 548	-------------

**Table 3:** Sequence of epitope 1806-1818 in chronic hepatitis C in relation to the presence of the HLA-DR1 allele.

HLA-DR1 positive(n = 8)		HLA-DR1 negative (n = 80)	
sequence	frequency	sequence	frequency
TLLFNILGGWVAA	7/8	TLLFNILGGWVAA	70/80
---L---------	1/8	---L---------	2/80
		----------M--	1/80
		-----V-------	1/80
		---------Z---	1/80
		-Z-----------	1/80
		------F------	1/80
		---C------L--	1/80
		----------L--	1/80
		-------X-----	1/80

The consensus sequence for the epitope aa 1806-1818 as used in the tetramer and functional assays is shown in full, and identity at a residue indicated by a dash. Z and X represent ambiguous residues within the bulk sequence.

Any mutation in epitope associated with DR1, p = NS

## Discussion

In this study we used a novel MHC Class II peptide tetramer for the detection of HCV-specific CD4+ T cells in HLA DR1+ patients with different stages and courses of HCV infection. This is the first HCV specific HLA class II tetramer to be tested and validated using epitope specific CD4+ T cell clones and we could demonstrate that this tetramer possesses similar sensitivity and specificity to HIV or influenza specific HLA class II tetramers characterized in a similar manner [8]–[11]. In addition, we took advantage of the fact that the HCV peptide used for the tetramer synthesis was highly targeted in our HLA-DR1+ study cohort. This limits the concern inevitably associated with the use of MHC-peptide tetramers in that they only detect responses against a single epitope with a single HLA restriction.

The major novel aspect of this study was the analysis of tetramer+ populations at early time-points after HCV infection, and most importantly, longitudinal analysis of the fate of these virus-specific populations in patients with a different clinical outcome of acute hepatitis C. The data clearly show that regardless of the long term clinical outcome, CD4+ T cells specific for this immunodominant epitope are present in all patients with acute disease. Interestingly, the frequency of tetramer+ populations in our cohort with acute hepatitis C was among the highest ever reported with HLA class II tetramers in viral infection studied ex vivo [9], [10], [12]. This may in part be due to the immunodominance of the epitope for HLA-DR1+ patients but it nonetheless demonstrates that, firstly, comparatively large numbers of HCV specific CD4+ T cells are induced during the early phase of HCV infection, and, secondly, the frequency of HCV specific CD4+ T cells is at least one log below the frequency of HCV specific CD8+ T cell populations.

The subsequent decline of tetramer+ CD4+ T cells mirrors the kinetics of HCV specific CD8+ T cells in acute hepatitis C that have been studied with HLA class I tetramers [19]. Importantly, whereas after spontaneous or treatment induced viral clearance tetramer+ CD4+ T cells levelled off at frequencies between 0.01% and 0.1%, the frequency further declined to very low (<0.01%) or undetectable levels in patients who developed chronic infection. This finding is consistent with a previous study employing an HCV specific HLA-DR4 tetramer that found a significant tetramer+ CD4+ T cell population in long-term recovered patients but not in patients with chronic hepatitis C [8]. Similarly, with the HLA-DR1 tetramer we found maintained levels of tetramer+ CD4+ T cells in three spontaneously recovered patients, one of whom had cleared HCV more than 20 years before, but only low frequencies in patients with chronic hepatitis.

Possibly the most informative facet of acute hepatitis C is the phase of transient viral control and subsequent viral recrudescence in the majority of patients who eventually develop chronic hepatitis C. It has previously been described that viral relapse is associated with a loss of T cell proliferative responses to HCV antigens [22], [23]. In addition, a recent case report using an HLA-DR4 tetramer showed that loss of proliferative capacity can occur without changes in the frequency of specific CD4+ T cells, indicating functional impairment rather than physical deletion [13]. In the current study, all three chronically evolving individuals went through a phase of transient viral control. In two of them tetramer+ CD4+ T cells remained detectable at comparable levels to the early phase indicating a loss of function whereas in the third patient tetramer+ CD4+ T cells became undetectable before viral recrudescence. Interestingly, these kinetics are in strong contrast to what has been observed in patients with acute HIV infection+/− short course antiretroviral therapy using HLA class II tetramers [10], [11]. Treatment-induced loss of HIV-RNA was associated with a decline in HIV specific tetramer+ CD4+ T cells, whereas recrudescence of HIV-RNA after treatment cessation induced a sharp increase in tetramer+ CD4+ T cells. Untreated patients also had responses which were sustained throughout the acute phase of infection. Long-term viremia may eventually be associated with a decline in tetramer+ cells; this, however, seems to be much slower than the decline in HCV-specific CD4+ T cells that we observed in patients with evolving chronic hepatitis C [11]. These data suggest that continued antigen re-encounter alone is insufficient to explain the loss of HCV specific CD4+ T cells during acute hepatitis C. Addressing compartmentalization of HCV specific CD4+ T cells to the liver is very difficult due to the lack of availability of liver biopsies from patients with acute hepatitis C. When we stained liver derived T cell lines from six HLA-DR1 positive patients with chronic hepatitis C we could not detect HCV specific CD4+ T cells although this issue certainly needs further study. Nor did we find any evidence for immune escape at an individual level (patients AC1 and AC2) or at a population level. Thus attractive hypotheses for the loss of viral control in acute hepatitis C could either be exhaustion of HCV specific T cells or a failure to establish stable memory CD4+ T cells during the phase of viral control which would explain why these cells do not expand when viremia returns and which is supported by the observation that in all three cases with evolving chronic hepatitis C CD4+ T cell function was lost before viral recrudescence.

In conclusion, using a novel HLA-DR1 tetramer complexed with an immunodominant CD4+ T cell epitope, we could show that HCV specific CD4+ T cells are induced in each patient with acute hepatitis C irrespective of outcome. Whereas spontaneous viral clearance is associated with long term maintenance of tetramer+ CD4+ T cells, early loss of function followed by depletion of tetramer+ CD4+ T cells is observed in patients with evolving chronic hepatitis C. The impact of such T cell loss may be very significant, undermining both CD8+ T cell-mediated and B cell mediated immune responses in the long term which can be associated with accentuated escape from CD8+ T cells as has been observed in model and natural infection [20], [24]. The mechanism behind this loss includes, as described above, excess stimulation by antigen, but an additional mechanism must also be sought to explain the differences from HIV. This may potentially be due to the specific environment where priming occurs, some activity of an HCV-derived protein such as core, defects in antigen-presentation, or a liver-specific “tolerance” effect. The new HLA class II tetramer technology will allow the further dissection of the mechanism behind this striking phenomenon which may hold the key to understanding HCV persistence.
